# Traction force with extracellular matrix mediated by cytoskeleton influences metastasis through SLC8A1 induced Wnt-β-catenin pathway in endometrial cancer

**DOI:** 10.1016/j.gendis.2023.101128

**Published:** 2023-09-26

**Authors:** Xingchen Li, Xin Xu, Jianliu Wang

**Affiliations:** a Department of Obstetrics and Gynecology, Peking University People's Hospital, Beijing 100044, China; b Department of Obstetrics and Gynecology, Beijing Chao-Yang Hospital, Capital Medical University, Beijing 100020, China

Endometrial cancer (EC) is one of the most prominent malignancies in gynecology. Unfortunately, despite these advancements in understanding EC pathogenesis, mortality rates have not decreased. Traction force (TF) refers to endogenous mechanical forces exerted by cells.^1^ These forces act on the extracellular matrix and the extracellular tumor microenvironment through focal adhesions, thereby regulating cell proliferation and migration. However, the TF characteristics of endometrial cancer cells and their heterogeneity remain unclear. SLC8A1, which encodes the Na^+^/Ca^2+^ exchanger, plays a crucial role in calcium homeostasis through calcium flux induced by cannabidiol and TRPV4 activation during mitophagy initiation. However, the regulatory mechanisms underlying SLC8A1 and its associated mechanical forces in EC remain elusive. In this study, we investigated the mechanical characteristics of different cell lines and primary EC cells. To the best of our knowledge, this is the first study to explore mechanical forces and identify a novel therapeutic target for EC.

To assess traction forces, cells were allowed to form monolayers on a substrate for 48 h prior to imaging. The cells were then mounted on a confocal microscope (Leica) and maintained at 37 °C with 5% CO_2_. Images were acquired at 10-to-20-min intervals over several hours. Lateral drift in the images was corrected using ImageJ software, and the computed displacements, traction stress, and monolayer contractile work were analyzed using the traction force microscopy (TFM) workflow. Detailed materials and methods are shown in the supplementary file.

In this study, we collected primary cells from tumor tissue samples of 24 patients with endometrial cancer, and their clinicopathological features are presented in Table S1. To study the impact of cell traction on invasion and metastasis ability, we selected four representative EC cell lines: Ishikawa, HEC-1-A, HEC-50B, and AN3CA, based on their tissue origin, pathological characteristics, and pathological grading. Single-cell traction tests revealed that HEC-1-A cells were used as the control group. As a result, when comparing the traction force among the four cell lines, no significant difference was observed between Ishikawa cells and HEC-1A cells (57.43 ± 19.52 Pa *vs*. 103.9 ± 27.40 Pa) (*P* > 0.05). However, the traction force of HEC-50B cells (37.34 ± 9.933 Pa) and AN3CA cells (17.97 ± 10.48 Pa) was significantly lower than that of HEC-1A cells (Fig. 1A). Functional analysis indicated that low TF cells had high metastatic ability (Fig. S1). Additionally, traction force measured by TFM indicated that cells in the progesterone-resistant groups exhibited lower TF values (Fig. 1B). To further investigate the associations between TF values and clinical features in EC patients, we then compared the TF values among patients with different clinicopathological characteristics (Table S2), revealing that high metastatic cells exhibited lower TF values (Fig. 1C, D). Meanwhile, TF values are also significantly diverse in patients with different MELF patterns (microcystic, elongated, and fragmented), histological types, cervical invasion, ovary involvement, tumor grades, and lymph-vascular space invasion (Fig. S2). Our previous study indicated the crucial role of SLC8A1 in mechanical-stimulus-induced progression in EC patients. Therefore, we investigated whether TF promotes metastasis through SLC8A1. The patient cohort was divided into low- and high-SLC8A1 groups based on the median threshold. We observed that the high expression of SLC8A1 was associated with worse survival compared with the low expression group (Fig. 1E; *P* = 0.02). Additionally, we explored the relationship between SLC8A1 and various clinical variables. The results revealed that EC patients with higher EC stage and grade were more likely to exhibit high SLC8A1 expression (Fig. S3, 4). We also validated this conclusion in patients in our center. Survival analysis demonstrated that high expression of SLC8A1 was associated with worse prognosis in EC patients in our hospital (Fig. 1F). Moreover, SLC8A1 expression was higher in patients with high grade, positive lymph node metastasis, positive peritoneal cytology, and positive lymph-vascular space invasion (Fig. S5). To further investigate the effect of SLC8A1 on the function of EC cells, we examined its expression in EC cell lines. The results revealed that SLC8A1 expression was highest in the Ishikawa cell line. To study the regulatory mechanism of SLC8A1 on the mechanical stimulus in EC cells, we explored the effect of SLC8A1 on metastatic ability and traction force in Ishikawa cells using single-cell traction tests. The outcomes revealed a significant reduction in the invasive ability of Ishikawa cells in the SLC8A1 low-expression group compared with the control group (Fig. S6). The results indicated a significant decrease in the traction force of endometrial cancer cells after SLC8A1 overexpression and an increase in the knockdown group (Fig. 1G). Based on the enrichment analysis of SLC8A1-associated differentially expressed genes, we investigated whether the downstream components of the Wnt-β-catenin pathway affected F-actin. Western blot analysis revealed decreased expression of Wnt, β-catenin, and F-actin in the si-SLC8A1 group. Furthermore, we confirmed that overexpression of SLC8A1 up-regulated these components, whereas their expression was significantly down-regulated after adding a Wnt inhibitor (Fig. 1F). Our data strongly suggested that SLC8A1 could promote the progression of EC and influence mechanical force, particularly F-actin, through the Wnt-β-catenin pathway.

**Figure 1:**
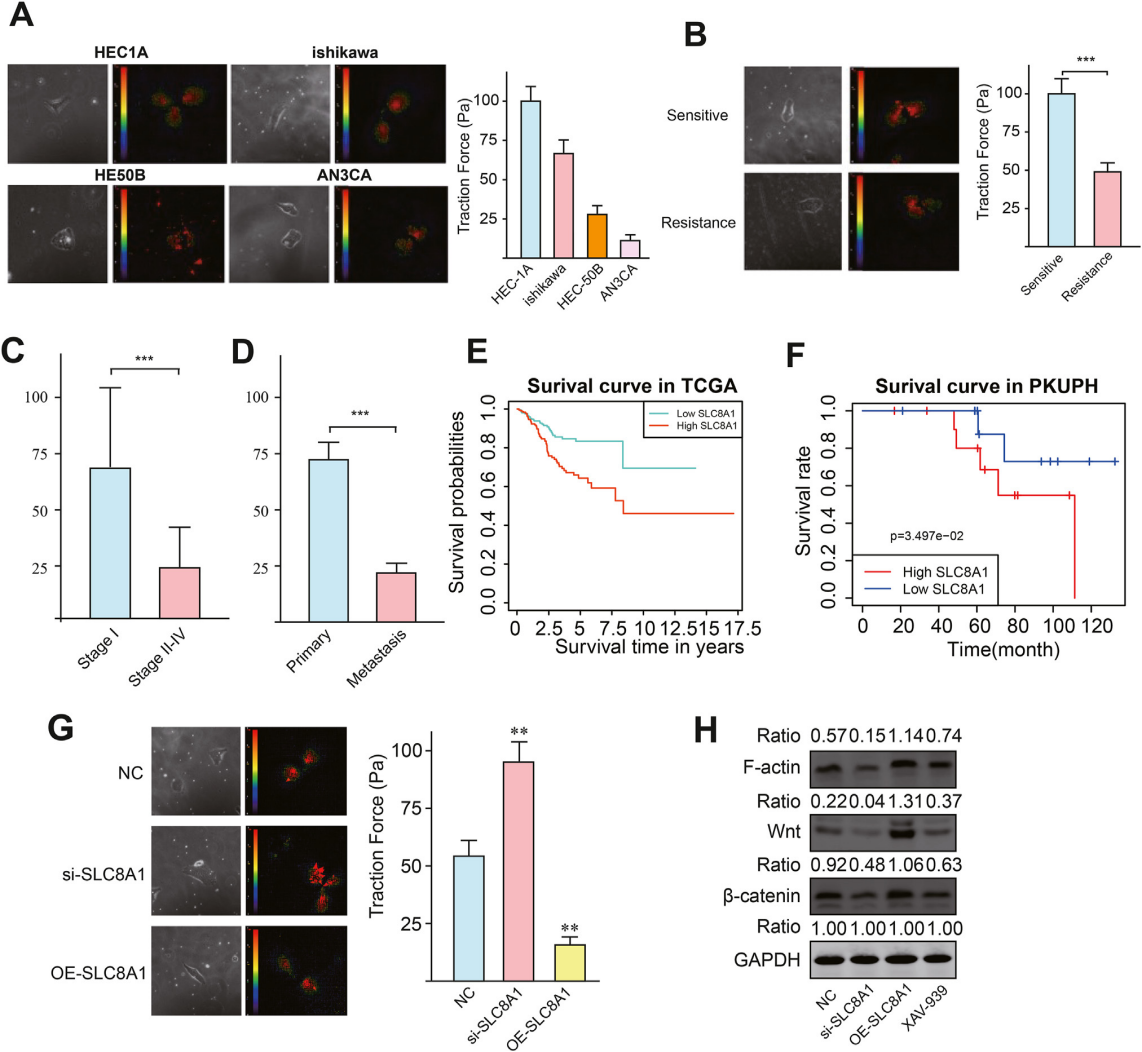
Functional analysis of traction force with characteristics and SLC8A1. **(A)** Heatmap of traction force microscopy (TFM) in four EC cell lines. **(B)** Heatmap of TFM in Ishikawa with different drug sensitivity. **(C)** Associations between traction force and stage. **(D)** Traction force between primary cancer and metastatic cancer. **(E)** Survival curve for patients with low and high expression of SLC8A1 in TCGA database. **(F)** Survival curve for patients with low and high expression of SLC8A1 in PKUPH. **(G)** Heatmap of TFM in Ishikawa with different expression levels of SLC8A1. **(H)** Effects of SLC8A1 on the Wnt-β-catenin signaling pathway measured by Western blot. ^∗^*P* < 0.05, ^∗∗^*P <* 0.01, ^∗∗∗^*P* < 0.001.

In this study, we investigated the biological function and clinical significance of mechanical force in the progression of EC by examining the regulation of SLC8A1 expression. Our findings provide evidence that mechanical forces, mediated by SLC8A1, are significantly associated with clinicopathological characteristics and the metastatic ability of EC. Primary cells or cell lines derived from EC, with varying invasive capabilities, can induce cytoskeletal deformation and alter the traction force of EC cells through SLC8A1. We demonstrated that SLC8A1 promotes EC migration by inducing F-actin reorganization through the Wnt-β-catenin signaling pathway. Specifically, we revealed the significant role of traction force in EC cell progression.

Tumor biomechanics has garnered considerable attention in recent years, as it contributes to our understanding of cancer cell behavior during tumor growth, metastasis, invasion, and adhesion. Adhesion forces between cells and the extracellular matrix are critical in multicellular organisms, with integrins serving as force transmitters between the intracellular cytoskeleton and the extracellular matrix. Studies have suggested that mechanical stimuli can induce malignant behavior in tumor cells by regulating cytoskeletal signaling pathways. The adhesion forces between cells and the extracellular matrix are associated with vinculin levels, and increased vinculin levels limit cell detachment, thereby impacting cell motility and migration.^2^ Stimulation from stiff extracellular matrix substrates triggers F-actin rearrangement, leading to decreased viscosity and increased migration of cancer cells. Leader cells generate high protrusive forces to overcome extracellular matrix resistance at the leading edge, and CDH3 was found to control leader cell protrusion dynamics through local production of integrin/focal adhesion function, as revealed by single-cell sequencing. Remodeling and reorganization of the cytoskeleton and cell matrix can alter traction force, thereby regulating cell movement in breast cancer.^3^ In our study, we identified the association between traction force and metastasis for the first time. Furthermore, we demonstrate that SLC8A1 acts as a key gene in cytoskeletal formation and deformation, promoting invasion and metastasis in EC cells.

Bioinformatics analysis has become a widely used approach to explore key genes involved in the pathogenesis and prognosis of various cancers. Nomogram models are increasingly used to predict cancer prognosis based on different clinicopathological features. In a previous study, our research group identified MMP12 as a potential prognostic biomarker and therapeutic target using integrated bioinformatics analyses and *in vitro* validation, highlighting the role of gene methylation and gap junctions in this process.^4^ In the current study, we focused on analyzing gene expression related to mechanical stimulation and identified SLC8A1 as a potential prognostic target in EC. Moreover, SLC8A1 is involved in a molecular signature with diagnostic and prognostic significance for overall survival and disease-free survival in oral squamous cell carcinoma. The fusion of SLC8A1 with its downstream intergenic region ALK showed an excellent response to ceritinib treatment and promising relapse-free survival.^5^

Our findings suggest that this effect is primarily mediated through alterations in the mechanical environment of tumor cells and the regulation of SLC8A1 expression. The data support the use of high SLC8A1 expression as a potential biomarker for evaluating metastatic capability in EC patients. Our results offer novel theoretical insights into EC metastasis and provide potential targets for its treatment.

## Ethics declaration

This study is approved by the Ethical Committee of Peking University People's Hospital, Beijing, China.

## Author contributions

Xingchen Li: conceptualization, software, investigation, data curation, and original drafting of the manuscript. Xin Xu: methodology, formal analysis, and data curation. Jianliu Wang: funding acquisition and writing-review & editing of the manuscript. All authors read and approved the final manuscript.

## Conflict of interests

The authors declare that they have no competing interests.

## Funding

This study is supported by the 10.13039/501100001809National Natural Science Foundation of China (No. 82103419, and 82230050) and the 10.13039/100006190Research and Development Fund of 10.13039/501100015083Peking University People's Hospital, Beijing, China (No. RS2021-05, and RDY2021-13).

## Data availability

The data underlying this article are available in the article and its online supplementary material.
