# Supplementary material for: Traction force with extracellular matrix mediated by cytoskeleton influences metastasis through SLC8A1 induced Wnt-β-catenin pathway in endometrial cancer
Source: Genes Dis. 2023 Sep 26;11(5):101128. doi: 10.1016/j.gendis.2023.101128 (PMC11176629; doi:10.1016/j.gendis.2023.101128)
Supplement: Multimedia component 1 [file mmc1.docx]

**Supplementary**

**Material and method**

**Tissue samples collection**

We collected a total of twenty-four EC and twenty normal endometrial tissue samples from patients at our institution. The samples were at our institution. The samples were classified according to the International Federation of Obstetrics and Gynecology (FIGO 2009) staging system and stored at -80°C. These samples were obtained from patients who underwent surgical resection at the Department of Obstetrics and Gynecology in Peking University People's Hospital (PKUPH) between January 2021 and January 2022. None of the patients had received preoperative chemotherapy, radiotherapy, or any other anti-tumor treatments. Two gynecologic pathologists verified all the diagnoses. The study received approval from the Ethics Committee of PKUPH, and informed consent was obtained from each patient.

**Cell culture and transfection**

We maintained four EC cell lines, namely AN3CA, Ishikawa, HEC1-A, and HEC-50B, in our laboratory. HEC-1A and HEC-50B cells were cultured in MYCOY'S 5A medium, while AN3CA and Ishikawa cells were cultured in DMEM/F12 medium. Both media were supplemented with antibiotics and 10% fetal calf serum (Gibco, USA). The cells were incubated in a 37 °C humidified incubator with 5% CO_2_. We obtained a negative control siRNA and SLC8A1-specific siRNA from GenePharm (Shanghai, China). Transfection of cells was performed using Lipofectamine RNAiMAX (Thermo, USA) according to the manufacturer's instructions. The cells were harvested after 48 hours for subsequent experiments.

**Transwell**

Cell migration was evaluated using transwell chambers (Corning, USA) with a pore size of 8 µm. Cells were suspended in serum-free medium (2.5 × 10^4^ cells) and incubated for 24 hours. The cells that migrated from the upper to the lower chamber were fixed with 4% paraformaldehyde (Beyotime) for 30 minutes and stained with crystal violet. The migrated and invaded EC cells were counted and quantified under an inverted light microscope by counting the cells in 10 random fields at ×100 magnification. The cells were photographed and quantified using ImageJ software.

**Wound-Healing Scratch Assay**

Target cells (5×10^6^) were plated in 6-well plates and incubated at 37°C until reaching 80-90% confluence. A wound line was created by scratching the cell monolayer with a sterile plastic tip (200μL). The suspended cells were removed with PBS, and the remaining cells were cultured in reduced serum MYCOY'S 5A or DMEM/F12 medium in a humidified 5% CO_2_ incubator at 37°C for 48 hours. Phase-contrast microscope images were captured, and three independent replicates were performed for each assay.

**Western blot**

Protein extraction was performed as previously described ^[19]^. After quantification, 50 µg of protein was separated using SDS-PAGE electrophoresis and transferred to NC membranes. The relative integrated density values (IDVs) were measured from the membranes using ImageJ software v.1.48, with GAPDH as a reference control.

**Immunofluorescence analysis**

Cells (2×10^5^ cells per well) were seeded on coverslips in a 6-well plate and cultured for 24 hours. The cells were then fixed with 4% paraformaldehyde for 30 minutes at room temperature, permeabilized with PBS containing 0.1% Triton X-100 for 10 minutes on ice and blocked with 1% fetal bovine serum (FBS). The coverslips were incubated overnight with anti-SLC8A1 antibody (1:1000 dilution), followed by washing with 0.1% PBS. Rhodamine phalloidin (100 nM) was added to the coverslips for 30 minutes in the dark, followed by another wash. DAPI (1:5000 dilution) was used for nuclear staining for 10 minutes, and the coverslips were immediately examined under a Leica SP8 Confocal Inverted Microscope (Leica, Germany). Image analysis was performed using ImageJ software, and the gray values of the nucleus and cytoplasm of each cell were calculated separately. Three repeats were performed for each experiment.

**Statistical analysis**

Data are presented as mean ± standard deviation (SD). Between-group differences were analyzed using student's t-test and one-way ANOVA. The relationship between traction values and clinicopathological parameters was determined using the χ2 test. Survival analysis was performed using the Kaplan-Meier method and log-rank test. Statistical analyses were conducted using SPSS 22.0 and GraphPad Prism 8.0 software (GraphPad, USA), and p < 0.05 was considered statistically significant.

**Supplementary Figures**

**Figure S1**

**
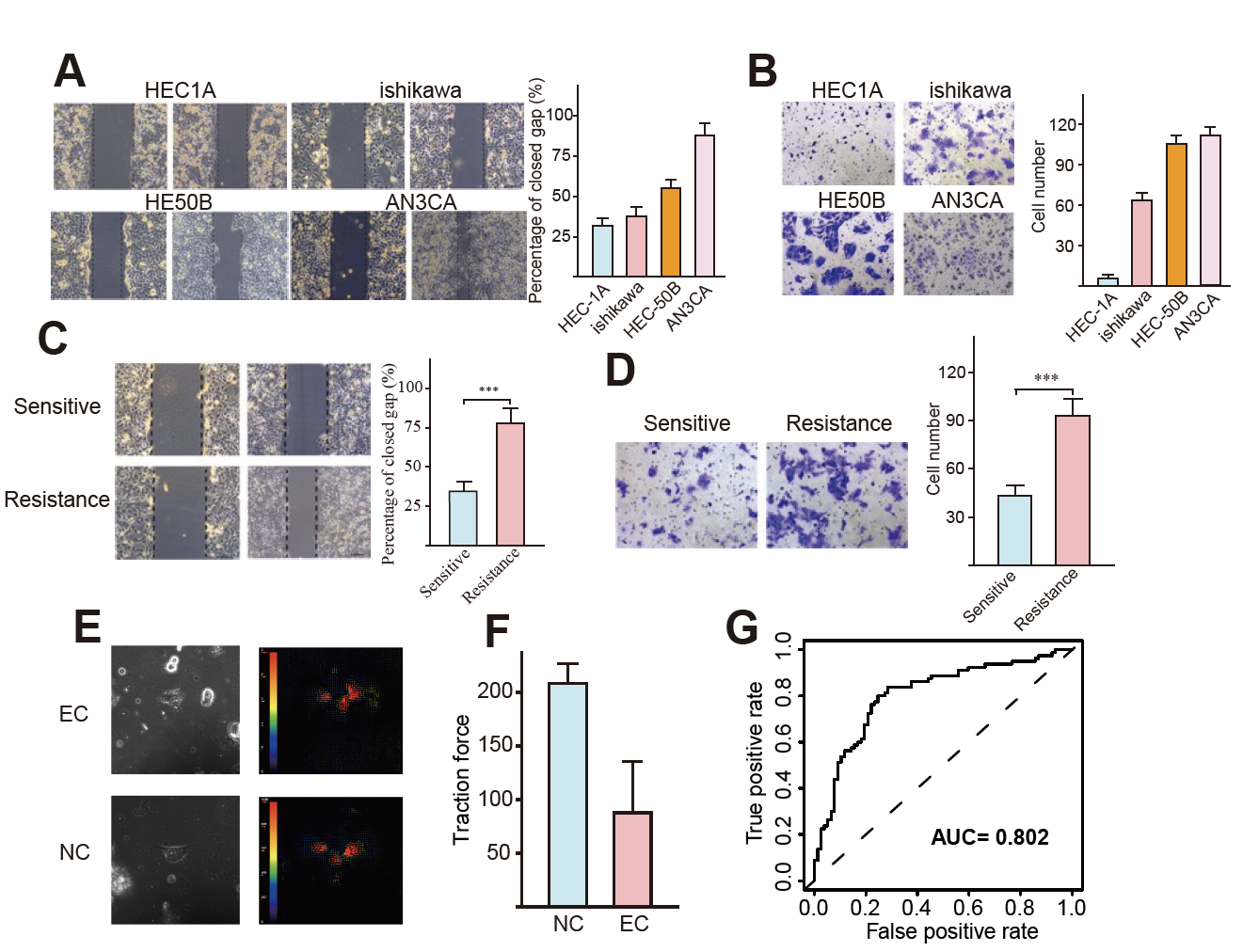
**

Functional analysis and traction force with different cells and drug sensitive. A. Gap closure analysis of different EC cell lines. B. Invasive ability of four EC cell lines by transwell analysis. C. Gap closure analysis of different drug sensitive. D. Invasive ability of ishikawa with different drug sensitivity by transwell. E. Heatmap of TFM in normal control and EC patients. F. Barplot of the TF values. G. ROC curve of predictive accuracy between TF and EC. EC, endometrial cancer; NC, normal control; TFM, Traction Force Microscopy

**Figure S2**

**
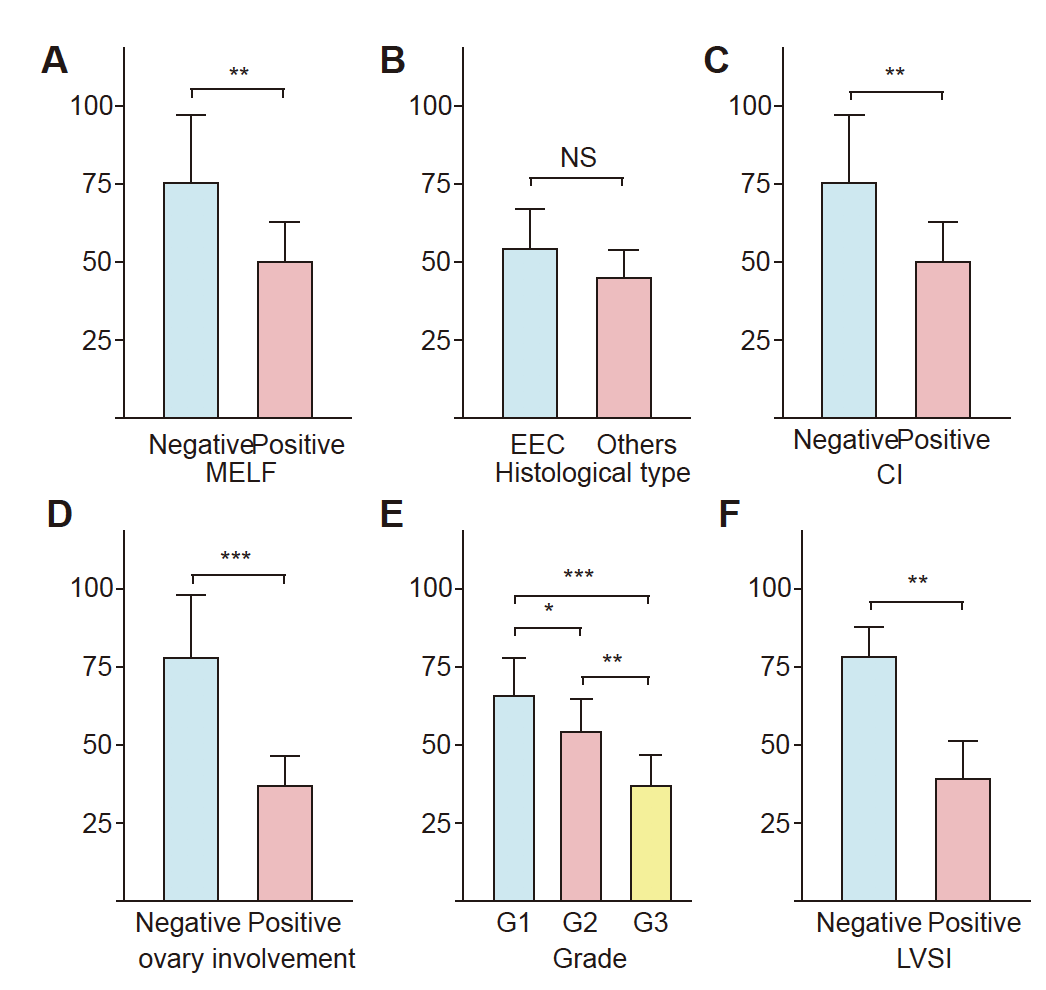
**

Associations between traction force and different clinicopathological characteristics. A. MELF. B. Histological type. C. CI. D. ovary involvement. E. tumor grade. F. LVSI.

EEC, endometroid endometrial carcinoma; G, grade; TF, traction force; FIGO, International Federation of Gynecology and Obstetrics; MELF, microcystic, elongated, fragmented; LVSI, lymph-vascular space invasion; CI, cervical invasion; LNM, lymph node metastasis.

**Figure S3**

**
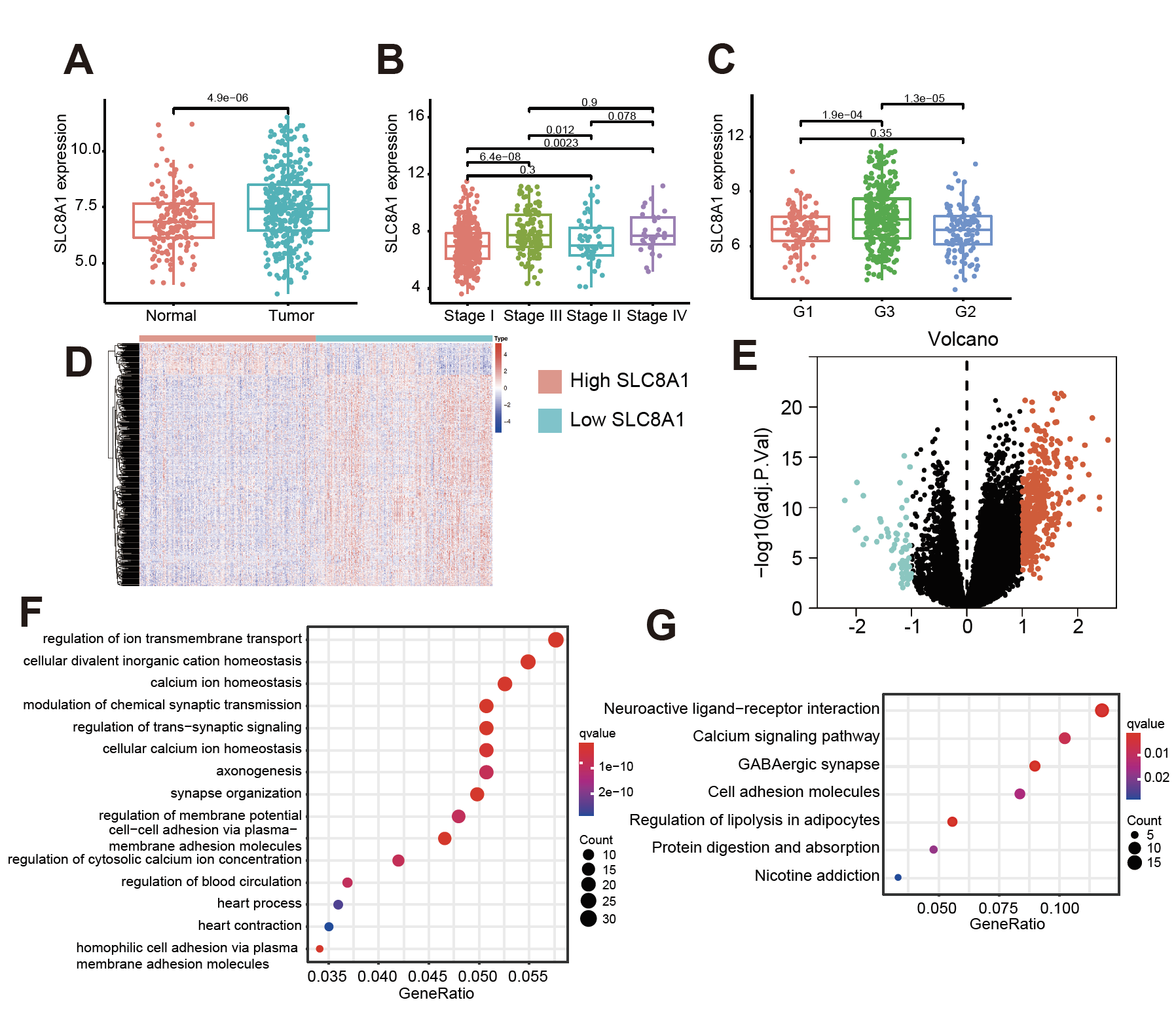
**

Bioinformatics analysis of SLC8A1 with EC patients in TCGA database. A-C. Expression of SLC8A1 in different tissue types, FIGO stage, and tumor grade. D. DEGs between low and high expression of SLC8A1 groups shown by heatmap. E. Volcano plot of DEGs. F-G. GO and KEGG analysis of DEGs.

DEGs, differentially expressed genes; GO, Gene Ontology, KEGG, Kyoto Encyclopedia of Genes and Genomes.

**Figure S4**

**
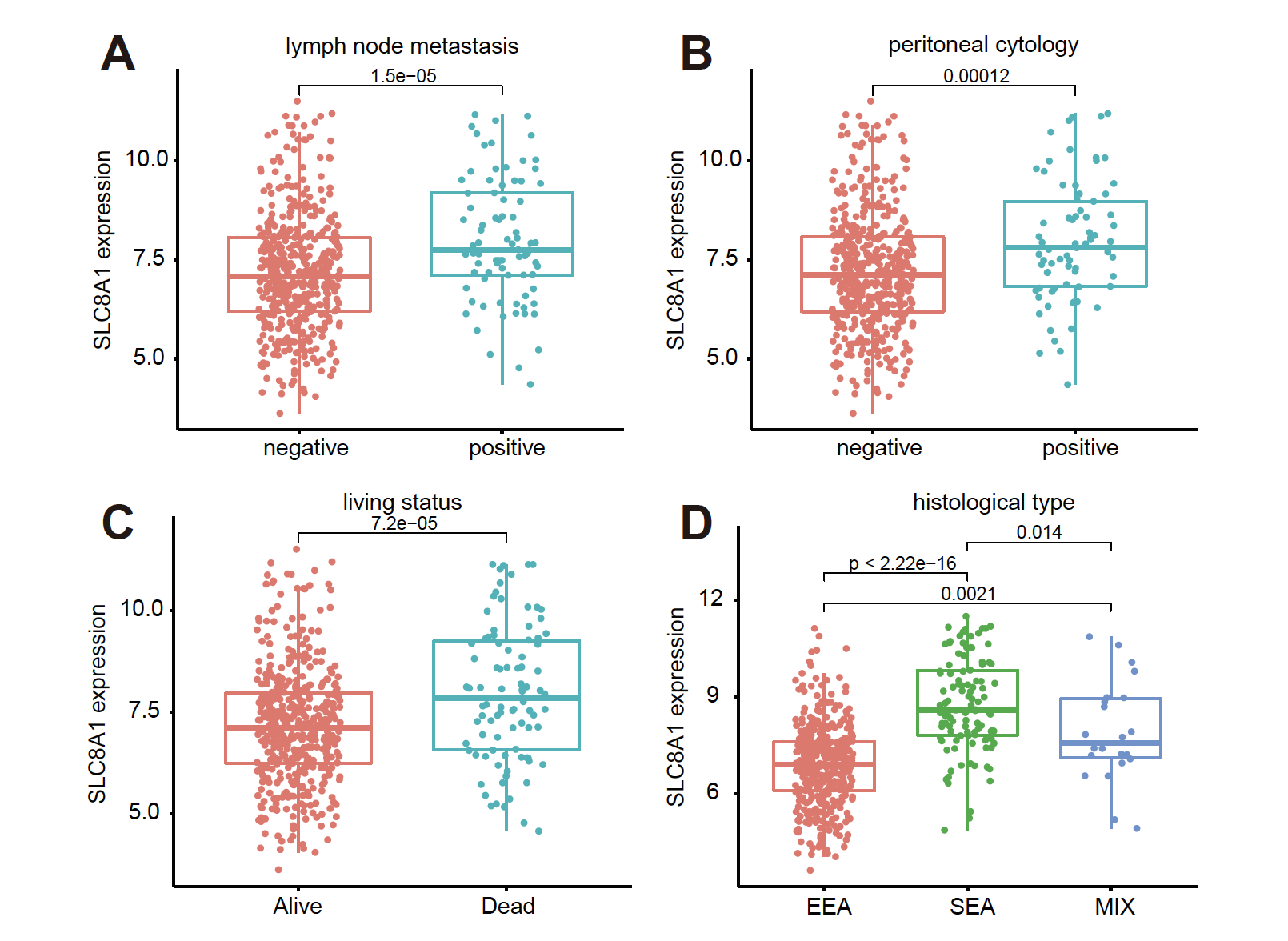
**

Expression of SLC8A1 in different clinicopathological characteristics. A. Negative and positive lymph node metastasis. B. Negative and positive peritoneal cytology. C. Alive and dead status. D. Different histological types.

**Figure S5**

**
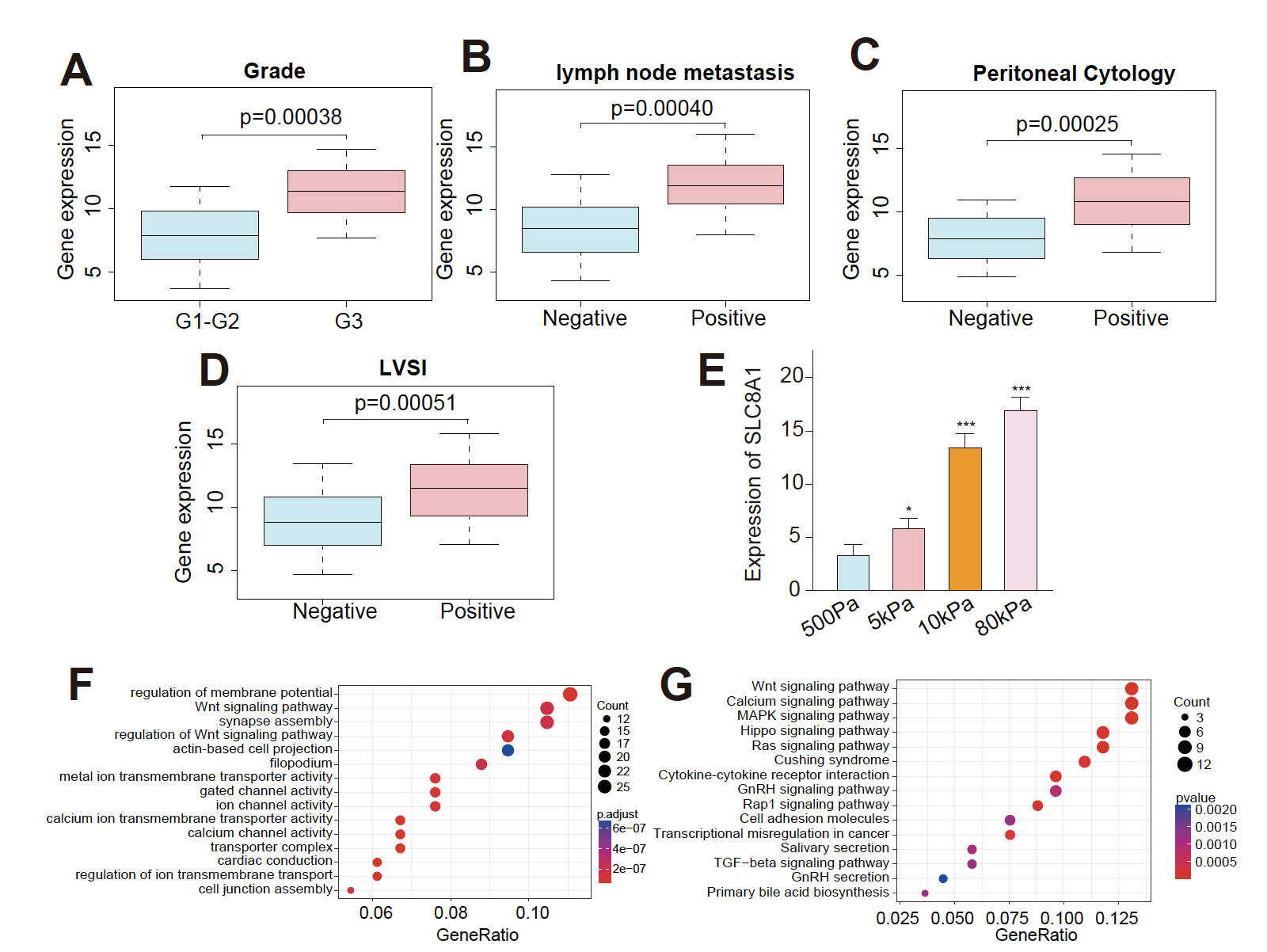
**

Validation of SLC8A1 function in PKUPH. A-D. Expression of SLC8A1 in different tumor grade, lymph node metastasis, peritoneal cytology, and LVSI. E. Expression of SLC8A1 in different stiffness of extracellular matrix. F-G. GO and KEGG analysis of DEGs of low and high expression of SLC8A1 subgroups.

**Figure S6**

**
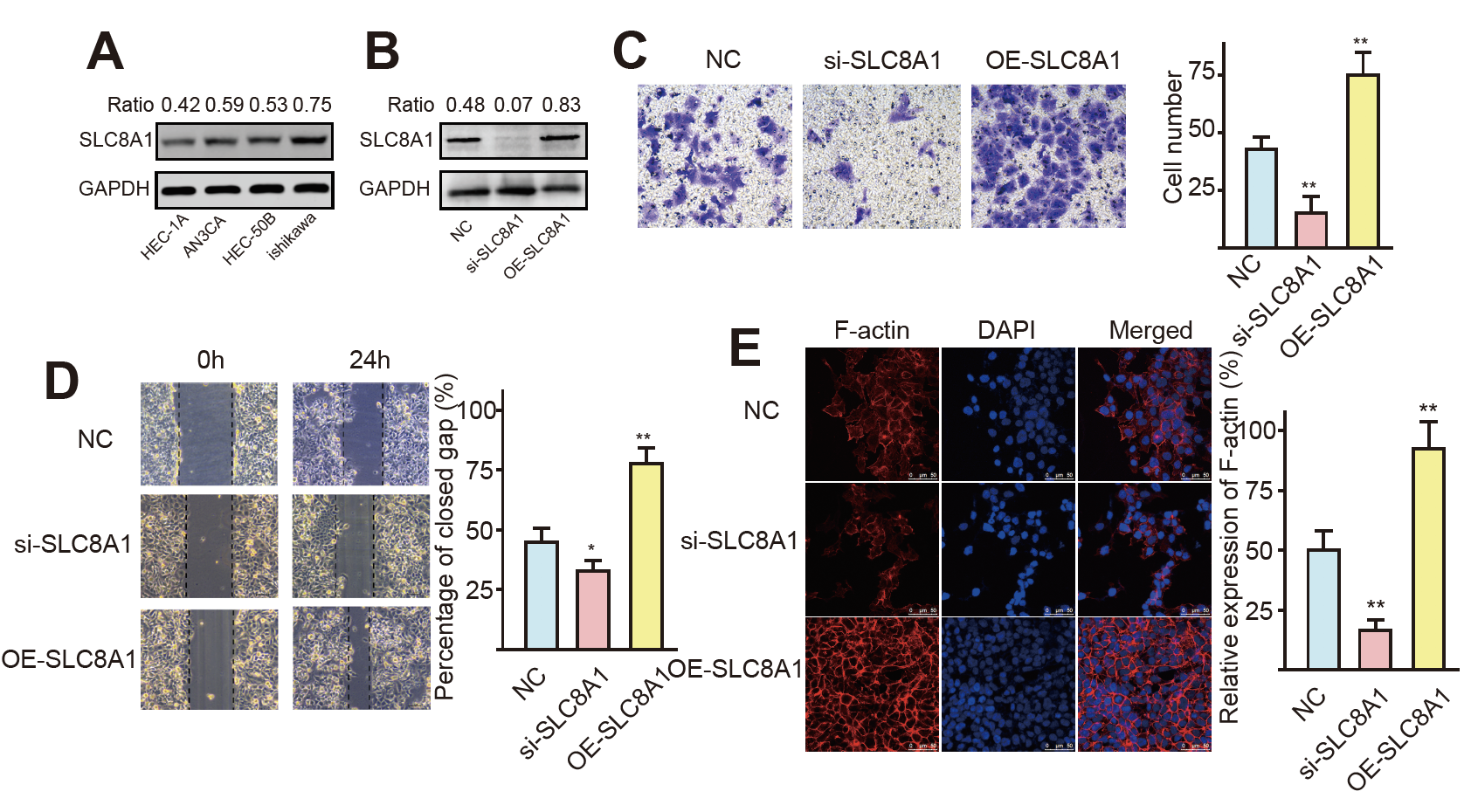
**

Function and downstream cytoskeleton for SLC8A1. A. Protein levels of SLC8A1 in four EC cell lines. B. The efficacy of SLC8A1 knockdown and overexpression in ishikawa cells measured through Western blotting. C-D. The invasion and metastasis ability of ishikawa in different expression of SLC8A1. E. expression of cytoskeleton (F-actin) in different SLC8A1 groups.

Table S1 Characteristics of patients in EC and control groups

| Category | EC | NC | *p*-value |
| --- | --- | --- | --- |
| Total | 24 | 20 | — |
| Age (year) | 59.54±9.179 | 38.95±12.79 | <0.0001 |
| Hight (cm) | 160.4±5.635 | 161.7±6.498 | >0.05 |
| Weight（kg）  BMI（kg/cm2）  TF（Pa） | 66.62±8.460  26.91±12.66  59.25±11.25 | 60.47±11.31  23.08±3.737  148.0±28.15 | <0.05  >0.05  <0.05 |

Footnote: EC, endometrial cancer; NC, normal control; BMI, body mass index; TF, traction force

Table S2 Clinicopathological features for 24 EC patients

| Variables | N（%） | Traction Force（Pa）  mean±SD | *p*-value |
| --- | --- | --- | --- |
| Histological type |  |  | 0.8768 |
| EEC | 22(91.67) | 61.22±57.23 |  |
| Other types | 2(8.33) | 38.99±2.043 |  |
| ER expression |  |  | 0.3551 |
| Negative | 2(8.33) | 77.62±52.59 |  |
| Positive | 22(91.67) | 57.71±56.15 |  |
| Grade |  |  | 0.1810 |
| G1 | 5(20.83) | 59.40±37.41 |  |
| G2 | 11 (45.83) | 56.27±27.48 |  |
| G3 | 8(33.33) | 35.15±13.80 |  |
| FIGO stage |  |  | 0.0033 |
| I | 17(70.83) | 71.24±61.62 |  |
| II-IV | 7(29.17) | 30.54±11.05 |  |
| MELF |  |  | 0.0010 |
| Negative | 16(66.67) | 48.26±58.60 |  |
| Positive | 8(33.33) | 74.25±26.74 |  |
| LVSI |  |  | 0.4585 |
| Negative | 13(54.17) | 58.89±70.22 |  |
| Positive | 11(45.83) | 59.93±32.44 |  |
| CI |  |  | 0.0412 |
| Negative | 20（83.33） | 56.12±28.04 |  |
| Positive | 4(16.67) | 31.73±10.87 |  |
| LNM |  |  | 0.4427 |
| Negative | 22（91.67） | 61.56±57.06 |  |
| Positive | 2（8.33） | 35.20±7.396 |  |
| Involving ovary and/or fallopian tube |  |  | 0.0388 |
| Negative | 20（83.33） | 73.97±62.61 |  |
| Positive | 4（16.67） | 35.01±9.023 |  |

Footnote: EEC, endometroid endometrial carcinoma; ER, estrogen receptor; G, grade; FIGO, International Federation of Gynecology and Obstetrics; MELF, microcystic, elongated, fragmented; LVSI, lymph-vascular space invasion; CI, cervical invasion; LNM, lymph node metastasis
